# Prognostic effect of pretreatment serum gamma-glutamyl transferase in urological malignancies: a systematic review and meta-analysis

**DOI:** 10.3389/fonc.2025.1597155

**Published:** 2025-06-25

**Authors:** Feifan Song, Shiqiang Su, Xueqiao Zhang, Xiongjie Cui, Chao Li, Shen Li

**Affiliations:** Department of Urology, Shijiazhuang People’s Hospital, Shijiazhuang, China

**Keywords:** urologic neoplasms, gamma-glutamyltransferase, prognosis, systematic review, meta-analysis

## Abstract

**Background:**

This study aimed ​​to investigate​​ the association between pretreatment serum gamma-glutamyltransferase (GGT) and survival outcomes in patients with urological malignancies, such as urothelial carcinoma (UCa), renal cell carcinoma (RCC), and prostate cancer (PCa).

**Methods:**

A comprehensive literature search was conducted in PubMed, Ovid, Web of Science, and the Cochrane Library up to December 2024. Survival outcomes were analyzed through the computation of merged hazard ratios (HRs) and 95% confidence intervals (CIs) using Stata 18.0 software.

**Results:**

Ten studies involving 2,817 patients were included in the final analysis. The results indicated that elevated pretreatment serum GGT demonstrated a significant association with poorer overall survival (OS) (HR = 3.32, 95% CI: 2.51-4.39), cancer-specific survival (CSS) (HR = 1.95, 95% CI: 1.26-3.04), and progression-free survival (PFS) (HR = 2.34, 95% CI: 1.72-3.17). Subgroup analyses stratified by cancer type demonstrated that elevated serum GGT served as a significant predictor of OS in UCa (HR 3.11, 95% CI 2.08-4.65), RCC (HR 3.51, 95% CI 2.27-5.43), and PCa (HR 3.61, 95% CI 1.51-8.62). Consistent associations were observed for CSS (Uca: HR 1.88, 95% CI 1.23-2.88) and PFS (Uca: HR 2.58, 95% CI 1.24-3.93; RCC: HR 2.00, 95% CI 1.28-3.13; PCa: HR 2.90, 95% CI 1.34-6.26). No significant publication bias was detected across the included studies.

**Conclusions:**

Pretreatment serum GGT served as an independent predictor of OS, CSS, and PFS in urological malignancies, suggesting that it may be a potential prognostic factor in clinical practice.

**Systematic review registration:**

https://www.crd.york.ac.uk/PROSPERO/view/CRD42025629976, identifier CRD42025629976.

## 1 Introduction

Urological malignancies, which comprise urothelial carcinoma (UCa), renal cell carcinoma (RCC), and prostate cancer (PCa), constitute a significant proportion of global cancer incidence, representing 12.6% of newly diagnosed cases in 2022 (1). These malignancies rank among the most frequently diagnosed cancers in the United States in 2024 (2). Recent advancements in targeted therapies, immunotherapies, and multimodal treatments have led to improvements in survival outcomes. However, the prognosis for individuals with advanced-stage conditions is still grim, primarily due to the high rates of recurrence and metastasis. ​​Notably, the 5-year survival rate associated with metastatic prostate cancer is a mere 31%, and in the case of advanced bladder cancer, the corresponding 5-year survival rate is as low as 14% (3). Additionally, among post-nephrectomy RCC patients, 74% of recurrences occur within 5 years after surgery (4). Given these challenges, identifying prognostic factors for survival and recurrence in urological malignancies are essential for guiding​​ personalized treatment strategies and optimizing patient outcomes.

Gamma-glutamyl transferase (GGT) plays a critical role in the metabolic pathway of glutathione (GSH), exerting appreciable influence on cellular redox homeostasis and oxidative stress responses, especially within cancer cells (5). Research indicates that elevated GGT levels are associated with increased all-cause mortality​​, ​​particularly cancer-related mortality (6). Moreover, there is a growing consensus that serum GGT may function as an autonomous prognostic indicator for multiple types of cancer, including breast cancer (7), hepatocellular carcinoma (8), pancreatic cancer (9), nasopharyngeal carcinoma (10), and ovarian cancer (11). Emerging studies have demonstrated ​​associations between serum GGT levels and clinical outcomes​​ in urological malignancies. However, the reported results were inconsistent. We intended to undertake a comprehensive analysis of the current literature to explore the predictive value of pretreatment serum GGT for urological malignancies.

## 2 Methods

### 2.1 Literature search process

Before conducting the meta-analysis, it was registered in PEROSPERO (CRD 42025629976). Moreover, the conduct of the study adhered to the PRISMA 2020 guidelines (12). ​​A systematic search strategy was developed and executed across four major biomedical databases​​ (PubMed, Ovid, Web of Science, Cochrane Library) ​​through December 10, 2024​​. The search strategy consisted of the following main terms: “gamma-Glutamyltransferase” (e.g., “gammaglutamyltransferase”, “GGTP”, “Glutamyl Transpeptidase”, “Transpeptidase, Glutamyl”, etc.), “Urologic Neoplasms” (e.g., “Neoplasms, Urologic”, “Carcinoma, Transitional Cell or Carcinomas, Transitional Cell”, “Carcinoma, Renal Cell”, “Prostatic Neoplasms”, “Urinary Bladder Neoplasms”, etc.), and “Prognosis” (e.g., “Prognoses”, “Prognostic Factors”, “Prognostic Factor”, “Factor, Prognostic”, etc.). Furthermore, we thoroughly examined the bibliographies of the identified studies to find any other eligible publications.

### 2.2 Inclusion and exclusion criteria

This comprehensive review synthesized evidence from original investigations assessing the predictive utility of pretreatment serum GGT measurements in patients with urological malignancies. Eligible studies met the following criteria (1): Patients had histopathologically confirmed urological malignancies, such as UCa, RCC, or PCa (2); The studies analyzed the correlation between pretreatment serum GGT levels and at least one survival endpoint, encompassing overall survival (OS), progression-free survival (PFS), and cancer-specific survival (CSS) (3); The studies directly reported or provided sufficient data to determine hazard ratios (HRs) and their corresponding 95% confidence intervals (CIs) for these outcomes (4); The studies were published in peer-reviewed journals.

Studies were excluded if they (1) were case reports, letters, meeting abstracts, or reviews (2); lacked sufficient data to calculate the HR and 95% CI (3); analyzed serum GGT as a continuous variable (4); were duplicates of previously published studies.

### 2.3 Data extraction and quality assessment

The systematic literature retrieval and critical appraisal were performed by two independent investigators. Discrepancies were resolved by a senior researcher. Comprehensive information was extracted from the included studies, covering key aspects such as authorship, publication details, demographic characteristics, study design parameters, tumor staging information, treatment modalities, GGT cutoff values with their determination methods, sources of HR with corresponding 95% CI, and evaluated survival outcomes. In addition, HRs and 95% CIs were derived either directly from univariate and multivariate Cox analyses or computed using available data from the literature. The inclusion of studies was assessed for quality employing the Newcastle-Ottawa Scale (NOS).

### 2.4 Data analysis

The present study employed Stata 18.0 (STATA Corporation, College Station) to conduct a thorough statistical analysis. The heterogeneity of study results was examined using the Higgins I^2^ statistic and Cochran’s Q test. In cases where substantial heterogeneity was detected (I^2^ > 50% and/or P < 0.10), a random-effects model was employed to derive the combined HR and 95% CI. Conversely, we used a fixed-effects model. Additionally, we performed subgroup analyses of categorical variables to investigate the association between serum GGT and OS. Publication bias was evaluated through a combination of funnel plot analysis and statistical tests, such as Begg’s and Egger’s test. Furthermore, we carried out sensitivity analysis by iteratively omitting single studies and recalculating the pooled estimates to assess the stability and dependability of the overall results.

## 3 Results

### 3.1 Study selection and key features

**Figure 1:**
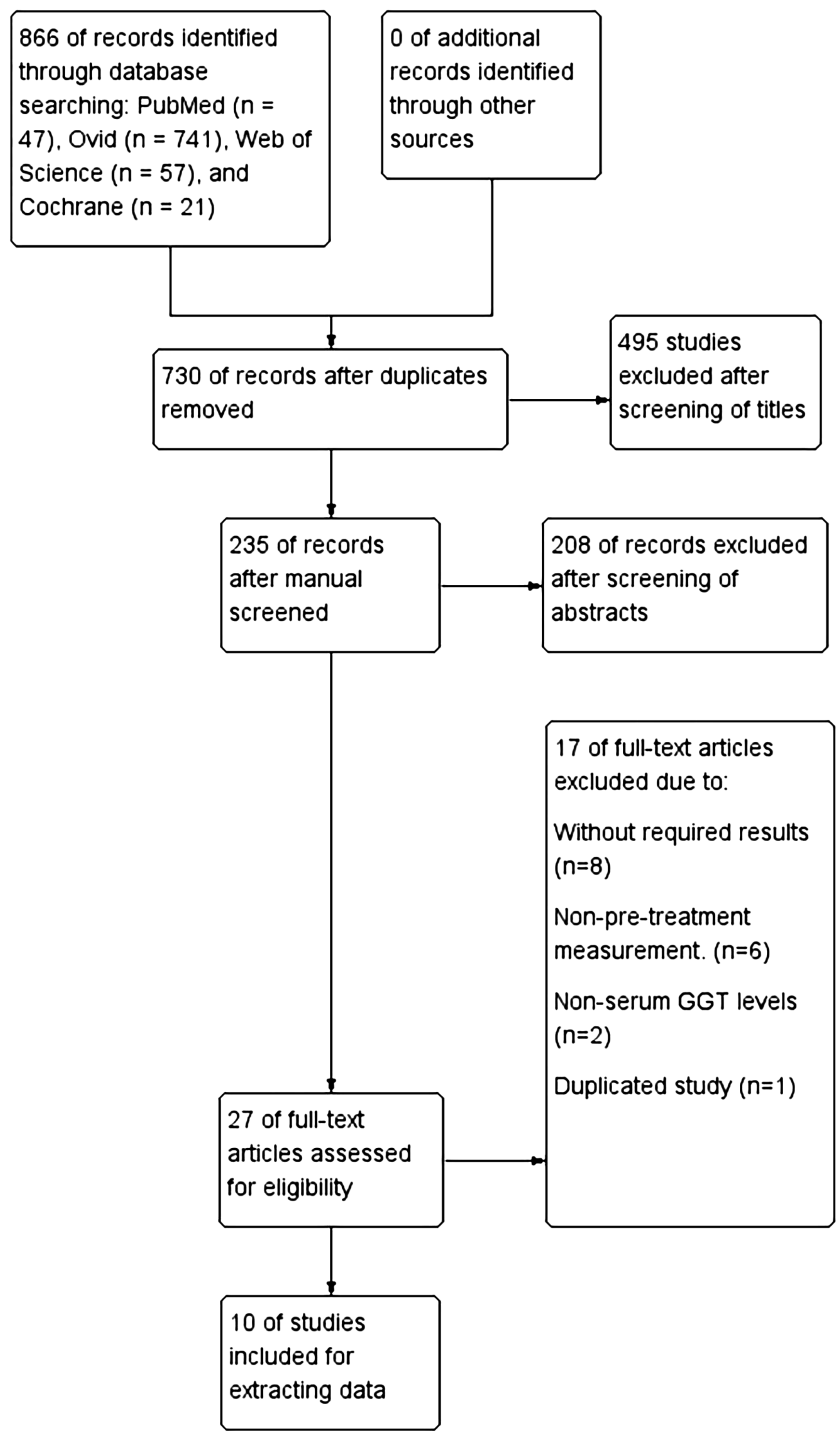
Flow diagram of the selection process for the included literature in the meta-analysis.

A flowchart illustrating the methodology for selecting the relevant studies was depicted in (**Figure 1**). We found 866 articles through a database search, and no additional literatures were identified through manual screening of reference lists from relevant articles. After removing 136 duplicate articles, reading the titles and abstracts resulted in the removal of 495 and 208 articles, respectively. 27 articles were evaluated by reading the full text. Among them, 8 were excluded due to the absence of prognostic-related data, 6 were excluded because they did not measure serum GGT levels before treatment, 2 were excluded as the study subjects were not serum GGT, and 1 was excluded because it was a duplicate. Ultimately, data from10 studies (13–22) were extracted for analysis.

All studies were retrospective designs published between 2014 and 2024. The majority of the study subjects were from Asian populations. These studies analyzed 2,817 patients. The median number of patients was 151 (IQR 82-324). The median age of participants was reported in 9 articles, ranging from 59 to 78 years. In all studies, 3 focused on UCa, 6 on RCC, and 1 on PCa. Most patients had undergone surgical treatment (**Table 1**).

**Table 1:** Main characteristics of the included studies.

Study	Year	Country	Study design	Case number	Age (Years)	Cancer type	Stage	Treatment	Cut-off (U/L)	Determine the cut-off value	Cox	Survival analysis	NOS
Buerk (13)	2024	Germany	RTP, SC	82	67 (40–82)R	RCC	Metastatic	Immunotherapy	34	Median	Mul	OS, PFS	8
Gakis (19)	2022	Germany	RTP, SC	324	66 (60–74)I	UCa	Non-metastatic	Surgery	55(men), 38 (women)	NR	Mul/Uni	OS, CSS	7
Su	2021	China	RTP, SC	268	63 (54–69)I	UCa	Non-metastatic	Surgery	40	ROC	Mul	OS, PFS, CSS	8
Ishiyama (14)	2021	Japan	RTP, SC	69	67 (54.00–73.00)I	RCC	Metastatic	Immunotherapy	49	Youden index	Mul/Uni	OS, PFS	7
Takemura (15)	2020	Japan	RTP, SC	146	66.5 (60.0–72.3)I	RCC	Metastatic	Targeted therapy	67.5	Reported	Mul	OS	8
Takemura (21)	2019	Japan	RTP, SC	50	78 (72–80)I	PCa	Metastatic	Targeted therapy	40	Martingale residuals	Mul	OS, PFS	8
Takemura (22)	2019	Japan	RTP, SC	101	70 (64−74)I	UCa	All	Surgery or Chemoradiation or Radiation	60	Martingale residuals	Mul	OS	8
Luo (16)	2017	China	RTP, SC	156	59.0 (51.0–66.0)I	RCC	Non-metastatic	Surgery	37.5	ROC	Mul	CSS	8
Dalpiaz (17)	2015	Austria	RTP, SC	700	65.4M	RCC	Non-metastatic	Surgery	40	ROC	Mul	CSS	8
Hofbauer (18)	2014	Austria	RTP, SC	921	64 (55–72)I	RCC	All	Surgery	34.5	RPA	Uni	CSS	7

NOS, Newcastle-Ottawa scale; RTP, retrospective; SC, single center; UCa, urothelial cancer; PCa, prostate cancer; RCC, renal cell carcinoma; ROC, receiver-operating characteristic; OS, overall survival; CSS, cancer-specific cancer; PFS, progression-free survival; NR, not reported; RPA, Recursive partitioning-based survival tree analysis; R, Median (range); I, Median (interquartile range); M Mean.

### 3.2 Summary analysis of OS

**Figure 2:**
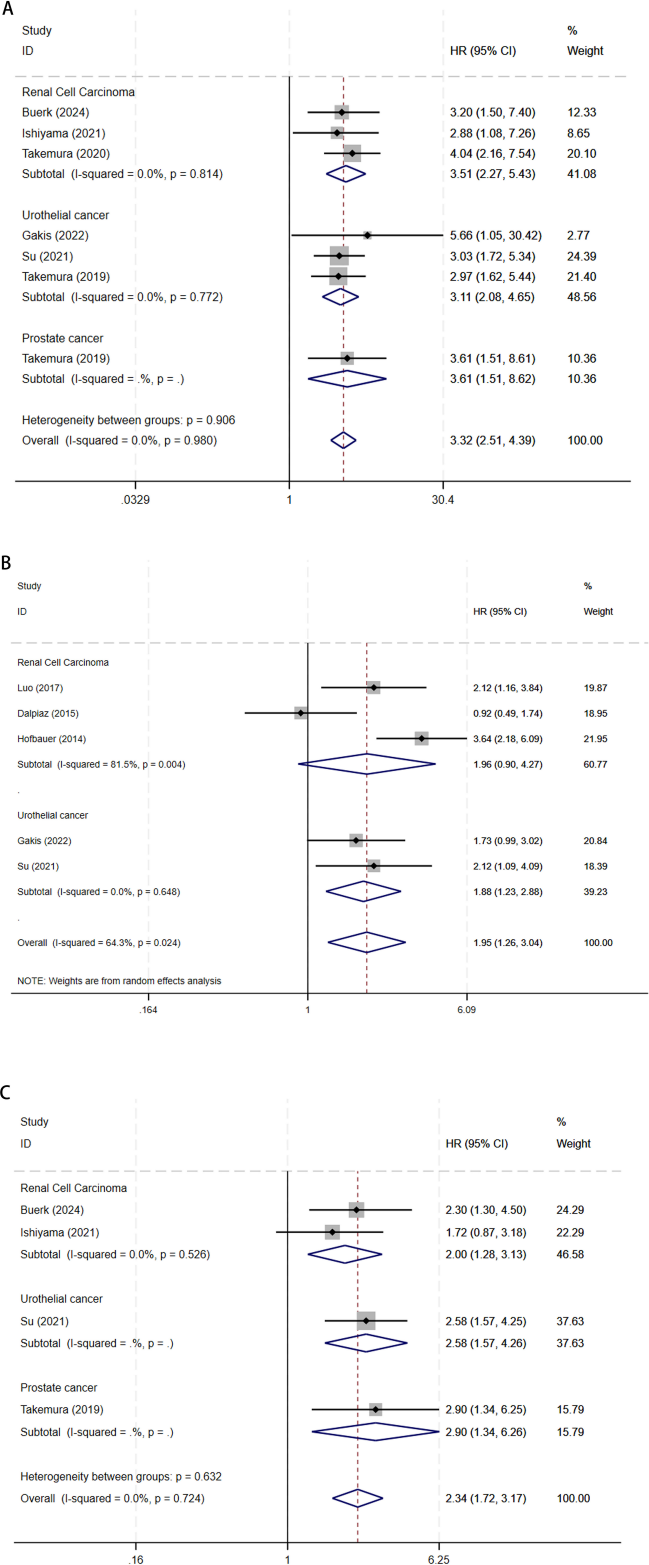
The forest plot of **(A)** OS, **(B)** CSS, and **(C)** PFS in elevated pretreatment serum GGT for urological malignancies.

Serum GGT and OS in urological malignancies: 7 articles studied the OS of urological malignancies (13–15, 19–22). A fixed-effects model was utilized for the analysis given the absence of substantial heterogeneity among the studies (I^2^ = 0.0%, P = 0.980). Our analysis demonstrated​​ that ​elevated serum GGT levels were significantly associated with worse OS in patients with urological malignancies (HR 3.32, 95% CI 2.51-4.39, P < 0.001). Stratified analysis by cancer type indicated that ​elevated serum GGT levels ​​​​were significantly associated with poorer OS in UCa (HR 3.11, 95% CI 2.08-4.65, P < 0.001), RCC (HR 3.51, 95% CI 2.27-5.43, P < 0.001), and PCa (HR 3.61, 95% CI 1.51-8.62, P = 0.004) (**Figure 2A**).

Besides tumor types, subgroup analyses of OS were also conducted based on year of publication, continent, sample size, and other variables, with considerable results still observed under these subgroup variables (**Table 2**).

**Table 2:** Results of subgroup analysis for OS.

Subgroup	Studies	HR (95% CI)	Heterogeneity		Z-value	P-value
			I2 (%)	P-value		
Overall survival						
Year of publication						
2020-2024	5	3.39(2.42-4.76)	0.0	0.918	7.06	<0.001
2014-2019	2	3.17(1.93-5.20)	0.0	0.718	4.54	<0.001
Continent						
Asia	5	3.28(2.42-4.44)	0.0	0.948	7.65	<0.001
Europe	2	3.55(1.73-7.31)	0.0	0.548	3.45	0.001
Site of carcinoma						
Urothelial cancer	3	3.11(2.08-4.65)	0.0	0.772	5.53	<0.001
Renal Cell Carcinoma	3	3.51(2.27-5.43)	0.0	0.814	5.63	<0.001
Prostate cancer	1	3.61(1.51-8.62)	NA	NA	2.89	0.004
Sample size						
>=100	4	3.36(2.40-4.71)	0.0	0.803	7.02	<0.001
<100	3	3.24(1.96-5.34)	0.0	0.942	4.60	<0.001
Cancer stage						
All	1	2.97(1.62-5.44)	NA	NA	3.52	<0.001
Non–metastatic	2	3.23(1.89-5.52)	0.0	0.490	4.27	<0.001
Metastatic	4	3.53(2.39-5.22)	0.0	0.937	6.33	<0.001
Treatment						
Surgery	2	3.23(1.89-5.52)	0.0	0.490	4.27	<0.001
Non-Surgery	5	3.35(2.42-4.66)	0.0	0.959	7.22	<0.001
Cut-off value						
>=60	2	3.45(2.23-5.37)	0.0	0.488	5.58	<0.001
<60	5	3.23(2.24-4.66)	0.0	0.964	6.27	<0.001
NOS score						
>=8	5	3.29(2.45-4.42)	0.0	0.957	7.88	<0.001
<8	2	3.39(1.48-7.77)	0.0	0.494	2.89	0.004

HR, hazard ratio; CI, confidence interval; NOS, Newcastle-Ottawa Scale.

### 3.3 Summary analysis of CSS

Serum GGT and CSS in urological malignancies: 5 articles studied the CSS of urological malignancies (16–20). The integrated findings highlighted that elevated serum GGT levels correlated with poorer CSS in urological malignancies (HR 1.95, 95% CI 1.26-3.04, P = 0.003), as identified through the random-effect model analysis (I^2^ = 64.3%, P = 0.024). CSS subgroup analysis revealed that elevated serum GGT levels were linked to poorer CSS in UCa (HR 1.88, 95% CI 1.23-2.88, P = 0.004), whereas no considerable association was observed in RCC (HR 1.96, 95% CI 0.90-4.27, P = 0.092) (**Figure 2B**).

### 3.4 Summary analysis of PFS

Serum GGT and PFS in urological malignancies: 4 articles studied the PFS of urological malignancies (13, 14, 20, 22). In the absence of considerable heterogeneity among studies (I^2^ = 0.0%, P = 0.724), the analysis using a fixed-effects model revealed that elevated serum GGT levels were related to a worse prognosis in terms of PFS among patients with urological malignancies (HR 2.34, 95% CI 1.72-3.17, P < 0.001). PFS subgroup analysis revealed that elevated serum GGT levels were linked to poorer PFS in UCa (HR 2.58, 95% CI 1.24-3.93, P < 0.001), RCC (HR 2.00, 95% CI 1.28-3.13, P = 0.002), and PCa (HR 2.90, 95% CI 1.34-6.26, P = 0.007) (**Figure 2C**).

### 3.5 Publication bias and sensitivity analysis

**Figure 3:**
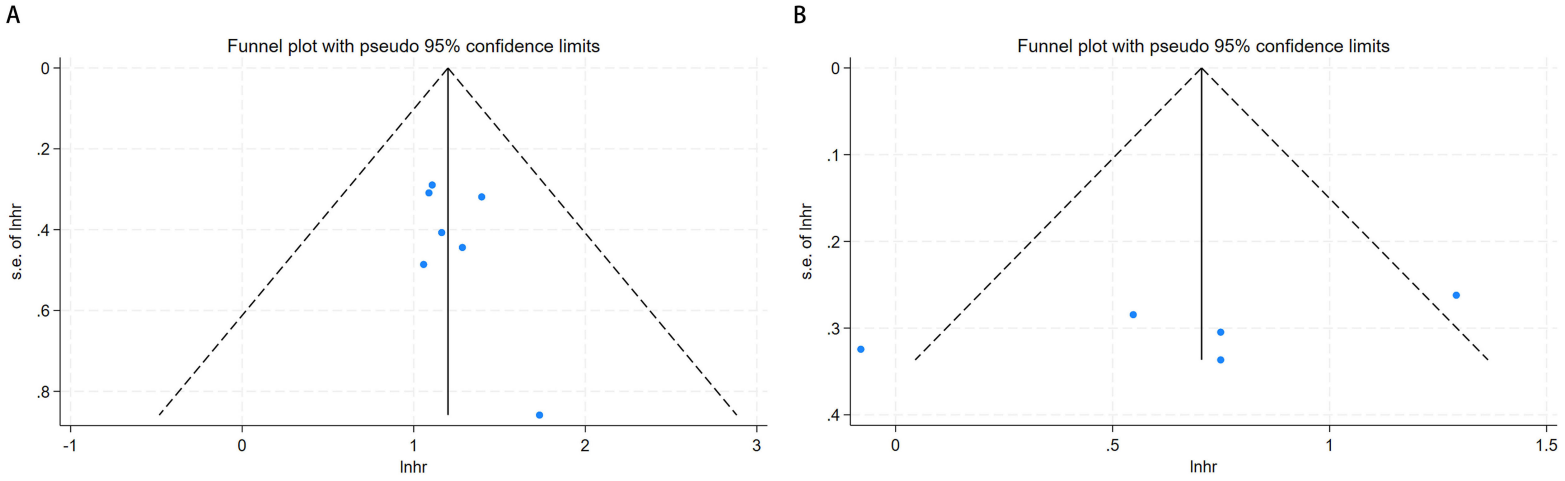
Funnel plot for publication bias. **(A)** correlation of pretreatment serum GGT with OS in urological malignancies; **(B)** correlation of pretreatment serum GGT with CSS in urological malignancies.

Visual inspection of the funnel plots for OS and CSS (**Figure 3**) revealed symmetrical distributions. Additionally, Begg’s and Egger’s tests quantitatively confirmed a minimal risk of publication bias for OS (P = 0.174 and 0.170) and CSS (P = 0.462 and 0.211).

**Figure 4:**
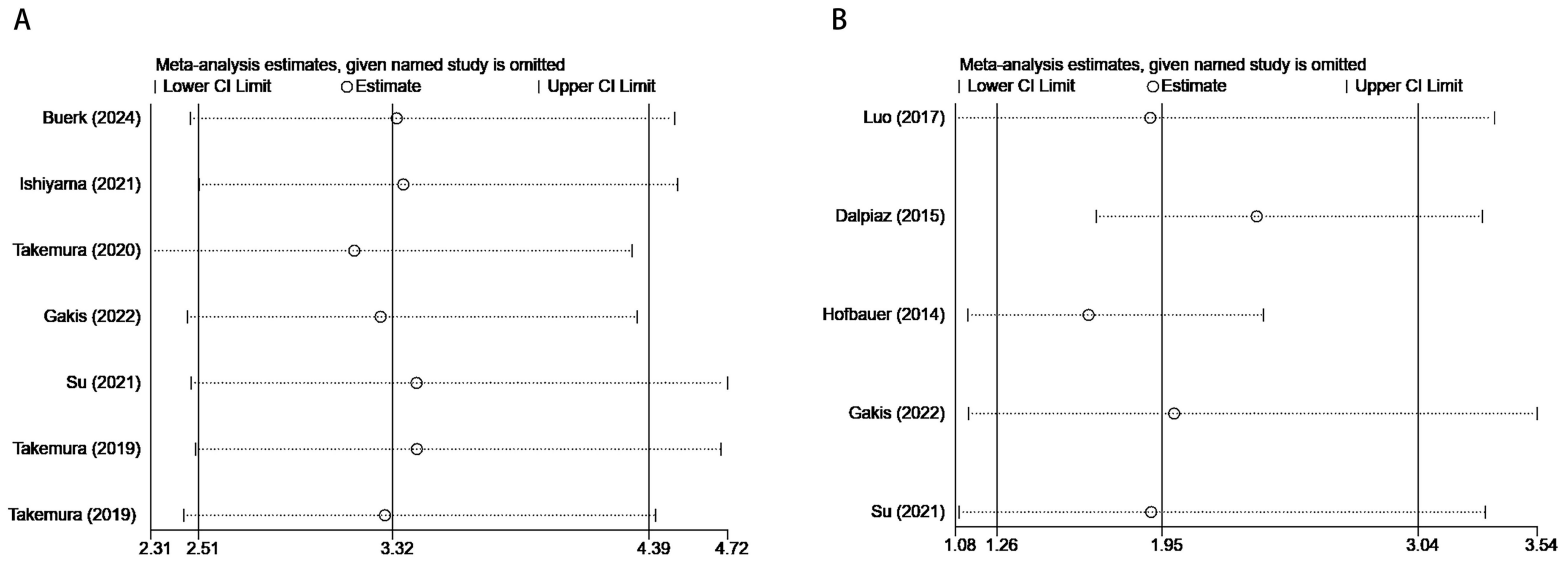
Results of sensitivity analysis. **(A)** correlation of pretreatment serum GGT with OS in urological malignancies; **(B)** correlation of pretreatment serum GGT with CSS in urological malignancies.

The stability of the meta-analytic results was examined through sensitivity analyses, focusing on the pooled HR and 95% CI for OS and CSS. Sequential exclusion of each study revealed that the pooled HR for the association between serum GGT and OS or CSS in urological malignancies remained consistent, ​​validating the robustness of the meta-analysis (**Figure 4**).

## 4 Discussion

Urological malignancies, such as UCa, RCC, and PCa, represent a major category of oncologic diseases with substantial global health impacts. PCa is the second most common cancer diagnosed globally and ranks fifth highest cancer-attributable deaths in males, with about 397,000 deaths documented in 2022. Bladder cancer, a principal subtype of UCa, ranks ninth in global cancer incidence, resulting in approximately 220,000 deaths annually. RCC has the 14th highest incidence and was estimated to contribute 15,600 deaths in 2022 (1). ​​The significant disease burden imposed by these malignancies, characterized by elevated incidence rates and considerable mortality​​, ​​underscores the critical need for identifying reliable prognostic factors. From our perspective, this research constitutes the inaugural full meta-analysis to ascertain the prognostic implication of serum GGT levels prior to therapy in urological malignancies.

The study was designed to methodically evaluate the function of serum GGT prior to therapy in the prognosis of urological malignancies by comprehensively reviewing the published studies on the subject and using meta-analysis. The meta-analysis on the basis of qualitative studies showed that the degree of pretreatment serum GGT elevation was considerably related to worse OS, CSS, and PFS of all the people who have UCa, worse OS and PFS of all the people who have RCC, and worse OS and PFS of all the people who have PCa. Subgroup analyses of OS based on different variables such as publication year, geographic region, sample size, tumor characteristics, treatment modalities, GGT cutoff values, and NOS score consistently maintained statistical significance under these subgroup variables. Our results were confirmed to be reliable and robust through publication bias assessments and sensitivity analysis. Serum GGT, a clinically utilized hematological marker, is straightforward and cost-effective to measure. Thus, serum GGT may operate as a valuable prognostic indicator in the management and clinical assessment of urological malignancies.

A recent systematic review investigated serum GGT as a prognostic factor for urological tumors. The authors included 8 articles and qualitatively concluded that elevated serum GGT levels were remarkably relevant to adverse prognosis in patients with urological malignancies (23). However, this study has several limitations. First, the search was restricted to PubMed and Cochrane, which likely resulted in an incomplete literature retrieval.​ Additionally, contradictory results among included studies may compromise the consistency and reliability of the conclusions. Second, most of the included studies focused on serum GGT levels, whereas Ramankulov et al. (24) investigated plasma osteopontin. Their study only briefly mentioned the relationship between serum GGT levels and survival without specifying survival endpoints. Therefore, our study did not include the work of Ramankulov et al. (24). To address these limitations, thorough searches were carried out across multiple databases, and the aggregated data were meta-analyzed to provide a quantitative conclusion. Consequently, our research offers the most current and comprehensive evidence regarding the prognostic significance of pretreatment serum GGT in urological malignancies.

While serum GGT is conventionally utilized to detect​​ hepatic abnormalities, biliary tract disorders, and impairment related to alcohol use, its role in tumor prognosis has garnered increasing attention (25). Recent studies propose​​ that GGT may have a key function in neoplasm development and resistance to anticancer drugs. Typically, patients with ​​elevated GGT levels exhibit significantly reduced survival durations across malignancies (26). Serum GGT is thus scientifically supported as a potential prognosis indicator in cancer patients. Nonetheless, the precise association between serum GGT levels and the clinical prognosis of urological malignancies remains to be elucidated, necessitating additional investigation. Emerging evidence indicates that GGT’s involvement in oxidative stress pathways substantially influences tumor metabolism in urological cancers. In highly metabolically active cancer cells, GGT levels are markedly upregulated under oxidative stress conditions (27). Tumor cells upregulate membrane-bound GGT to facilitate the absorption of GSH, a key antioxidant, from the bloodstream and surrounding tissues, thus acquiring extra cysteine and cystine to restore intracellular GSH levels (28). However, in certain specific cases, GGT may also promote oxidative reactions. Continuous oxidative stress contributes to genomic instability and regulates the regulation of tumor progression (29). Moreover, studies have shown that GGT induction can be triggered by inflammatory mediators, including tumor necrosis factor and interleukin (30, 31). GGT is crucial in the metabolic pathway involving the inflammatory mediator leukotriene C4 (32). In summary, elevated serum GGT levels may influence the progression and outcomes of urological malignancies by regulating redox homeostasis and participating in inflammatory processes.

While this study provides valuable insights, several limitations warrant acknowledgment. Firstly, the incorporated studies were exclusively single-center and retrospective in design, thus inherently vulnerable to recall bias and residual confounding. Furthermore, Some HRs and corresponding 95% CIs were derived from univariate analyses lacking covariate adjustment, potentially introducing uncontrolled confounding biases. Secondly, dependence solely on published literature may engender publication bias due to potential underrepresentation of studies with non-significant findings. Thirdly, despite comprehensive search strategies, the paucity of articles reporting PFS outcomes compromises the robustness of corresponding conclusions. Finally, the predominance of Asian studies and the lack of validation in multi-center, ethnically diverse populations may limit the generalizability of our findings.

## 5 Conclusion

Elevated pretreatment serum GGT levels constitute an independent prognostic factor for shorter OS, CSS, and PFS durations​​ in urological malignancies. Collectively, pretreatment serum GGT levels may be a valuable indicator for informing clinical management strategies in urological malignancies.

## Data Availability

The original contributions presented in the study are included in the article/supplementary material. Further inquiries can be directed to the corresponding author/s.
